# Secretion of the Phosphorylated Form of S100A9 from Neutrophils Is Essential for the Proinflammatory Functions of Extracellular S100A8/A9

**DOI:** 10.3389/fimmu.2018.00447

**Published:** 2018-03-13

**Authors:** Véronique Schenten, Sébastien Plançon, Nicolas Jung, Justine Hann, Jean-Luc Bueb, Sabrina Bréchard, Eric J. Tschirhart, Fabrice Tolle

**Affiliations:** ^1^Calcium Signalling and Inflammation Laboratory, Life Sciences Research Unit, University of Luxembourg, Belvaux, Luxembourg

**Keywords:** neutrophils, S100A8/PhosphoA9, inflammation, cytokines, Toll-like receptor 4

## Abstract

S100A8 and S100A9 are members of the S100 family of cytoplasmic EF-hand Ca^2+^-binding proteins and are abundantly expressed in the cytosol of neutrophils. In addition to their intracellular roles, S100A8/A9 can be secreted in the extracellular environment and are considered as alarmins able to amplify the inflammatory response. The intracellular activity of S100A8/A9 was shown to be regulated by S100A9 phosphorylation, but the importance of this phosphorylation on the extracellular activity of S100A8/A9 has not yet been extensively studied. Our work focuses on the impact of the phosphorylation state of secreted S100A9 on the proinflammatory function of neutrophils. In a first step, we characterized the secretion of S100A8/A9 in different stimulatory conditions and investigated the phosphorylation state of secreted S100A9. Our results on neutrophil-like differentiated HL-60 (dHL-60) cells and purified human neutrophils showed a time-dependent secretion of S100A8/A9 when induced by phorbol 12-myristoyl 13-acetate and this secreted S100A9 was found in a phosphorylated form. Second, we evaluated the impact of this phosphorylation on proinflammatory cytokine expression and secretion in dHL-60 cells. Time course experiments with purified unphosphorylated or phosphorylated S100A8/A9 were performed and the expression and secretion levels of interleukin (IL)-1α, IL-1β, IL-6, tumor necrosis factor alpha, CCL2, CCL3, CCL4, and CXCL8 were measured by real-time PCR and cytometry bead array, respectively. Our results demonstrate that only the phosphorylated form of the complex induces proinflammatory cytokine expression and secretion. For the first time, we provide evidence that S100A8/PhosphoS100A9 is inducing cytokine secretion through toll-like receptor 4 signaling.

## Introduction

Neutrophils are among the first cells to be recruited to inflammatory sites with a crucial role in pathogen killing through the deployment of sophisticated antimicrobial strategies, including production of reactive oxygen species (ROS) by the nicotinamide adenine dinucleotide phosphate-oxidase (NADPH oxidase), release of cytotoxic products during the degranulation process and formation of neutrophil extracellular traps (NETs). Neutrophils are also able to secrete an impressive array of autocrine and paracrine mediators, including cytokines and chemokines, which can recruit other immune cells and regulate their functions.

Activation of neutrophils was initially recognized as the result of the presence of pathogens, but lately it has become clear that this inflammatory response can also be triggered by endogenous ligands known as damage-associated molecular pattern (DAMPs) or alarmins (1). Recently, S100A8 and S100A9 have been characterized as DAMPs, which are released by activated phagocytes such as neutrophils and monocytes (2, 3). These two proteins are members of the S100 multigene subfamily of cytoplasmic EF-hand Ca^2+^-binding proteins and are abundantly expressed in the cytosol (~40% of total proteins) of circulating neutrophils and present both intra- and extracellular activities. Intracellularly, S100A8 and S100A9 are known to regulate NADPH oxidase activity (4, 5), the major source of ROS in neutrophils. Extracellularly, high concentrations of S100A8/A9 are found at local sites of inflammation or in the serum of patients with inflammatory diseases (e.g., rheumatoid arthritis, cystic fibrosis, or inflammatory bowel disease) (3, 6, 7) and represent sensitive inflammation biomarkers (8) strongly associated with disease level. Moreover, it is now evident that S100A8/A9 are not only biomarkers for inflammatory conditions but also have a pathogenic function (9). Plasma S100A8/A9 concentration has been correlated with blood neutrophils counts and both associated with the incidence of coronary events and cardiovascular mortality (10). Further investigation is however necessary to establish the exact role of S100A8/A9 in the development of cardiovascular diseases. On the one hand, S100A8/A9 elevation resulting of the increase of neutrophils in the blood could promote atherogenesis *via* the recruitment of neutrophils in the arterial wall (11). On the other hand, S100A8/A9 have been described to exert a repellent effect on neutrophils (11). There is growing evidence that S100A8/A9 have a dual role in the inflammation process. The balance between pro- and anti-inflammatory functions of S100A8/A9 seems in part rely on the oxidative state of S100A8/A9. Oxidation of S100A9 was found to be required for its chemotactic effect and antimicrobial activity (12, 13). Additionally, it has been suggested that posttranslational modifications of S100A8 induced by oxidase-producing oxygen derivatives may switch their biological properties from a proinflammatory toward an anti-inflammatory pattern preventing excessive damage to host tissue by scavenging oxidants (14, 15).

Once released in the extracellular environment, S100A8 and S100A9 contribute to the amplification of the inflammatory process, through a plethora of functions, either by activation of neutrophils (autocrine mode of action) or by other inflammatory cell types (paracrine mode of action) (3). S100 proteins exert their activities on neighboring cells through the engagement of pattern recognition receptors, such as toll-like receptor 4 (TLR4) or the receptor for advanced glycation end products (RAGE) signaling (2, 16, 17). First evidence that RAGE was able to interact with S100 family members came from S100A12 (18). Later, S100A9 and S100A8/A9 have been shown to bind immobilized human recombinant RAGE (19). The activation of tissue-resident sentinel cells by S100A8/A9 can for example result in the enhancement of leukocyte recruitment to inflammatory sites (20) or transport of arachidonic acid to target cells (21).

S100A8/A9 secreted by phagocytes are of importance in the pathophysiology of many inflammatory diseases (3), however, the mechanisms by which S100A8 and S100A9 are released are still not completely resolved. S100 proteins do not possess the leader sequence typical for transport *via* the classical endoplasmic reticulum/Golgi pathway and thus, are released by an alternative secretory pathway. In this view, Rammes et al. have suggested that this non-classical S100A8/A9 secretion is an energy-dependent process, which depends on an intact microtubule network and PKC activation, at least in monocytes (22). In neutrophils however, the mechanism related to S100A8/A9 secretion could be different since S100A8/A9 have been detected in NETs (23).

Formation of Ca^2+^-dependent S100A8/A9 heterotetramers is likely a prerequisite for their biological activity (24), which seems to be regulated by the posttranslational modification of S100A9. Indeed, S100A9 can be phosphorylated at Threonin 113 by p38MAPK in activated neutrophils and monocytes (25, 26) and this phosphorylation contributes to microtubule reorganization and phagocyte migration (2, 26) but also to the regulation of the neutrophil NADPH oxidase (5). However, the phosphorylation state of secreted S100A9 as well as the impact of this phosphorylation on the extracellular activities of S100A8/A9 has not yet been investigated.

Therefore, our main objective was to determine the phosphorylation state of secreted S100A9 and to evaluate the impact of this phosphorylation on the extracellular proinflammatory activity of the S100A8/A9 complex.

Our data indicate that S100A9 is released in a phosphorylated form from differentiated HL-60 (dHL-60) cells and purified human neutrophils probably by NETosis. The phosphorylated form of S100A9 in the complex, and not the non-phosphorylated form, is able to induce the secretion of cytokines such as tumor necrosis factor alpha (TNFα) or interleukin (IL)-6 through TLR4 signaling. We were thus able to demonstrate that the phosphorylation of S100A9 modulates the proinflammatory response of neutrophils to S100A8/A9.

Our study provides the first evidence that the proinflammatory functions of secreted S100A8/A9 could be directly attributed to the phosphorylation of S100A9. Given the multiple roles of S100A8/A9 and their importance in the development of many inflammatory diseases, this could constitute a significant advance in our understanding of the pathogenesis of these diseases and represent an opportunity for more specific and efficient treatment.

## Materials and Methods

### Reagents and Antibodies

RPMI-1640 medium, l-glutamine, penicillin, and streptomycin were purchased from Life Technologies (Gent, Belgium) and fetal bovine serum was purchased from Lonza (Verviers, Belgium). fMLF, phorbol 12-myristoyl 13-acetate (PMA), and diphenyleneiodonium (DPI) were obtained from Sigma-Aldrich (Bornem, Belgium). All other chemicals were of analytical grade and obtained from Merck (Darmstadt, Germany). The physiological salt solution (PSS) used had the following composition: NaCl 115 mM, KCl 5 mM, KH_2_PO_4_ 1 mM, glucose 10 mM, MgSO_4_ 1 mM, CaCl_2_ 1.25 mM, HEPES-Na 25 mM, pH 7.4. The following antibodies were used in this study: anti-calgranulin clone CF145 (Santa Cruz Biotechnology, Dallas, TX, USA); anti-S100A8/A9 antibody clone Mac387 (DAKO, Glostrup, Denmark), anti-calgranulin B (S100A9) clone B-5 (Santa Cruz Biotechnology, Heidelberg, Germany); anti-MRP8 (S100A8) clone EPR3554 (Abcam, Cambridge, UK); antimyeloperoxidase (anti-MPO) antibody clone 2C7 (Thermo Fischer Scientific, Erembodegem, Belgium); anti-neutrophil elastase antibody (reference 411001, Calbiochem, Overijse, Belgium), and anti-TLR4 neutralizing antibody (reference AF1478, R&D Systems, Oxfordshire, UK).

### Cell Culture

The promyelocytic cell line HL-60 (ATCC #CCL-240) was grown in RPMI-1640 medium supplemented with 10% heat-inactivated fetal bovine serum, l-glutamine (2 mM), streptomycin (100 μg/mL), and penicillin (100 U/mL). The cells were kept at 37°C, in a humidified atmosphere with 5% CO_2_ and subcultivated twice a week. The differentiation of HL-60 cells toward neutrophil-like cells was induced by addition of DMSO (1.3% v/v) for 4.5 days (27).

### Purification of Human Neutrophils

For the purification of human neutrophils, peripheral blood of healthy volunteers was collected into EDTA-containing tubes (Vacutainer^®^, BD Biosciences, Erembodegem, Belgium). Samples were collected following the good clinical and ethical practices, which were approved by the Ethics Review Panel of the University of Luxembourg according to the guidelines of the “Comité National d’Ethique de Recherche” from Luxembourg.

The neutrophils were isolated by dextran sedimentation prior to centrifugation on a Ficoll-Hypaque gradient. Remaining erythrocytes were then lysed for 10 min in red blood cell lysis buffer (155 mM NH_4_Cl, 10 mM KHCO_3_, 0.1 mM EDTA, pH 7.4) (28).

Neutrophils were washed and resuspended in PBS (NaCl 137 mM, KCl 2.7 mM, Na_2_HPO_4_ 10 mM, KH_2_PO_4_ 1.8 mM, pH 7.4). Purity of isolated neutrophils was assessed by flow cytometry (BD FACSCanto II flow cytometer, BD Biosciences) using following selection markers: CD66b-FITC, CD16-BV510, CD45-PECy7, CD14-APC, and CD49d-PE (BD Biosciences) as selection markers on 10.000 events in the gated population of homogenous (FSC-A versus SSC-A), single (SSC-A versus SSC-H), and living cells (negative for Sytox Blue staining; Invitrogen, Ghent, Belgium). Purified neutrophils are positive for CD66b, CD16, and CD45 while being negative for CD49d and CD14, allowing for the discrimination from eosinophils or monocytes, respectively. Only preparations containing more than 98% of neutrophils were used for further experiments.

### Production of Recombinant Proteins

Human S100A8 and S100A9 were recombinantly expressed in *Escherichia coli* and purified using affinity column. Briefly, *E. coli* BL21(DE3) bacteria were transformed with pGEX-5X-1 expression vector containing S100A8 or S100A9 cDNA. Recombinant GST-S100A9 and GST-S100A8 were induced and then purified using glutathione sepharose column. The immobilized GST-protein was eluted by Xa protease cleavage directly in the column. Then, recombinant S100A8 and S100A9, without GST-tag, were obtained and used for the validation of the anti-PhosphoS100A9 antibody.

### Purification of S100A8/A9 and S100A8/PhosphoA9

S100A8 and S100A9 were purified from human granulocytes as described previously (29). Granulocytes were prepared from human buffy coats as described above, lysed by sonication and the cytosolic fraction was obtained by centrifugation (200,000 *g*, 1 h, 4°C). The protein concentration of the supernatant were adjusted to 3 mg/mL with buffer A (Tris 50 mM, EDTA 1 mM, EGTA 1 mM, dithiothreitol, 1 mM, pH 8.5) and a large portion of irrelevant proteins was removed by ammonium sulfate (70% w/v) precipitation for 2 h at 4°C. Precipitated samples were then subjected to 10,000 *g* centrifugation for 30 min at 4°C. The supernatant, enriched in S100A8/A9 protein complex was dialized overnight at 4°C against buffer A. The final purification step is done by FPLC using an anion exchange column (HiTrap Q XL, GE Healthcare Life Sciences, Diegem, Belgium). Proteins were eluted with a gradient 0–0.4 M NaCl at a flow rate of 1 mL/min. The elution of pure S100A8/A9 and S100A8/PhosphoA9 fractions is obtained at NaCl concentrations of 0.12–0.135 and 0.25–0.27 mM, respectively. The purity of proteins was greater than 95%, as verified by SDS-PAGE under reducing conditions and visualized by Coomassie stain and mass spectrometry analyses.

### S100A8/A9 ELISA

For the measurement of S100A8/A9 secretion, 5 × 10^5^ cells/mL were stimulated in PSS under different conditions and the supernatants were collected. S100A8/A9 concentration in the supernatants was determined by ELISA (BMA Biomedicals, Augst, Switzerland), after appropriate dilution of the samples, according to the manufacturer’s recommendations.

### Sample Preparation for Western Blot

For the validation of the Phospho-specific S100A9 antibody, dHL-60 were stimulated by 100 nM fMLF for 10 min and the cells were lysed in a Triton lysis buffer (15 mM Tris, 150 mM NaCl, 1% Triton, SigmaFast protease inhibitor cocktail and phosphatase inhibitor cocktail from Sigma). 75 μg of this lysate were then treated for 15 min or 1 h with calf intestinal phosphatase (CiP, 75U) in order to dephosphorylate S100A9. Loading buffer (Tris 63 mM pH 6.8; 2% SDS; 10% glycerol; 0.1% β-mercaptoethanol) was added to different lysates as well as recombinant S100A8 and recombinant S100A9 and all samples were heated for 5 min at 97°C. For cell culture supernatants, samples were treated as follows: supernatants of control or stimulated cells were collected and concentrated by centrifugation in an Amicon^®^ Ultra 3K Centrifugal Filter Device. Loading buffer (Tris 63 mM pH 6.8; 2% SDS; 10% glycerol; 0.1% β-mercaptoethanol) was added to the concentrated supernatants and the samples were heated at 97°C for 5 min.

### Western Blot

Samples were run on a Tris-Tricine gel (10% acrylamide) and the proteins were then electrotransferred to a PVDF membrane (Merck-Millipore, Overijse, Belgium). Immunodetection of the cell lysate was performed using the polyclonal rabbit antibody directed against Phospho-S100A9 (custom-made by Thermo-Fischer) or the monoclonal antibody Mac387 (DAKO) and horseradish peroxidase-conjugated donkey antirabbit or rabbit antimouse secondary antibodies. The immunodetection was visualized by the non-commercial enhanced chemiluminescence solution as described by Haan and Behrmann (30).

For immunodetection of purified S100A8/A9 and S100A8/PhosphoA9 fraction, the anti-PhosphoS100A9 antibody was used alone or preincubated 1 h at room temperature with 1 or 4 μg/mL of immunogenic peptide (CHKPGLGEG[pT]P) as well as the antibody B-5 (Santa Cruz Biotechnology) and EPR3554 (Abcam). The secondary antibodies antirabbit Alexa Fluor 680 (Life Technologies) and antimouse IRDye 800 (LI-COR Bioscience, Lincoln, NE, USA) were used for fluorescent Western blot detection with the Odyssey Infrared Imaging system (LI-COR Biosciences).

For the immunodetection of concentrated supernatants, the anti-PhosphoS100A9 antibody was used as well as the antibody Mac387 (DAKO). The secondary antibodies antirabbit IRDye 800 CW and antimouse IRDye 680 LT (LI-COR Bioscience) were used for fluorescent Western blot detection with the Odyssey Infrared Imaging system (LI-COR Biosciences).

### Quantification of NETs by Plate Assay

Neutrophil extracellular traps were quantified using a fluorescent plate assay. dHL-60 cells or freshly purified neutrophils were cultured in a black 96-well plate. After indicated treatments, the cell impermeable DNA binding Dye Sytox blue (ThermoFischer, Ghent, Belgium) was added to the wells at a concentration of 0.5 μM to detect extracellular DNA. Fluorescence was measured on a Clariostar plate reader (CLARIOstar, BMG LABTECH, Ortenberg, Germany). The baseline fluorescence of the media with the dye was subtracted from all readings and the results were expressed according to the fluorescence of the non-stimulated control.

### NET Detection by Confocal Microscopy

Neutrophil extracellular trap formation was visualized by staining of extracellular DNA, MPO, and human neutrophil elastase. Briefly, (dHL-60) or freshly purified neutrophils were washed and 4 × 10^5^ cells were placed on polylysin-coated coverslips and placed for 1 h at 37°C + 5% CO_2_. Then, cells were stimulated by different compounds for 4 h and fixed for 15 min at 4°C in PBS containing 3.7% w/v paraformaldehyde and 2% w/v sucrose. The cells were then washed and permeabilized with PBS containing 0.1% saponin and 0.2% BSA and blocked with human IgGs for 15 min. Anti-MPO, antielastase, or isotype control antibodies were applied to the cells for 30 min in a humidity chamber at RT. The cells were washed three times and then incubated with a fluorophore-labeled secondary antibody (DyeLight-conjugated anti-mouse antibody, cyanin3-conjugated anti-rabbit antibody from Jackson ImmunoResearch, Sufolk, UK) together with DAPI 0.5 μM for 30 min in a humidity chamber at room temperature. Cover slips were washed four times and mounted in Moviol/DABCO and fluorescent images were acquired using the laser-scanning confocal microscope LSM510 (Carl Zeiss, Zaventem, Belgium) with a 40x objective and a multitrack configuration.

### Quantitative Real-time PCR

First-strand cDNA was prepared from 0.5 μg of total RNA, by adding the reaction buffer, 0.2 μg random hexamers, 20U RNAsin Ribonuclease inhibitor (Promega), 0.5 mM dNTP, and 40 U GoScript reverse transcriptase (Promega). Reverse transcription was performed as follows: 5 min at 25°C, 60 min at 42°C, and 10 min at 70°C. PCR primers for *interleukin 1 alpha* (*IL-1α*), *interleukin 1 beta* (*IL-1β*), *IL-6, TNF*α*, chemokine (C-C motif) ligand 2, 3, and 4* (*CCL2, CCL3*, and *CCL4*), and *chemokine (C-X-C motif) ligand 8* (*CXCL8 alias IL-8*) as well as the primers for the three housekeeping genes *beta 2 microglobulin* (*B2M*), *Beta-actin* (*ACTβ*), and *beta-glucuronidase* (*GUSβ*) were designed based on published sequences in GenBank (see Table 1 for primer sequences). qPCRs were performed using the SYBR^®^ Select master Mix (Thermo Fischer Scientific, Erembodegem, Belgium) in a QuantStudio 12K Flex real-time PCR machine (Thermo Fischer Scientific). The cycling protocol was as follows: 3 min at 50°C and 3 min at 95°C followed by 40 cycles of 3 s at 95°C and 30 s at 60°C. Relative quantification of the different mRNAs, expressed in arbitrary units (AU), were calculated using three reference genes (*ACTβ, GUSβ, B2M*) and the Vandesompele method, based on the geometric averaging of multiple internal controls (31).

**Table 1:** Sequence of primers used in RT-qPCR.

Gene	Forward (5′–3′)	Reverse (5′–3′)
*IL1-α*	AATCTGGATGAAGCAGTGAAA	CATCTTGGGCAGTCACATAC
*IL1-β*	CTACAGCTGGAGAGTGTAGA	GAACTGGGCAGACTCAAAT
*IL-6*	CACACAGACAGCCACTCACC	AGTGCCTCTTTGCTGCTTTC
*TNFα*	CCCATGTTGTAGCAAACCCT	TGAGGTACAGGCCCTCTGAT
*CCL2*	CTGCTCATAGCAGCCACCTT	GCTTCTTTGGGACACTTGCT
*CCL3*	CAGATTCCACAGAATTTCATAGC	CTTGGTTAGGAAGATGACAC
*CCL4*	CACCAATGGGCTCAGAC	GCTGCTGGTCTCATAGTAATC
*CXCL8*	ACAAGAGCCAGGAAGAAAC	AACTGCACCTTCACACAG
*ACTβ*	GCCCTGAGGCACTCTTCCA	TGTTGGCGTACAGGTCTTTGC
*B2M*	AAGCAGCATCATGGAGGTTT	TGGAGCAACCTGTCAGATA
*GUSβ*	CAAGAGCCAGTTCCTCATCA	TTGAAGTCCTTCACCAGCAG

### Measurement of Cytokine Secretion by Cytometric Bead Array (CBA)

Density of dHL-60 cells was adjusted to 0.5 × 10^6^ cells/mL per stimulatory condition for subsequent quantitative measurement of cytokine secretion. Supernatants were collected and immediately used for CBA (BD Biosciences, Erembodegem, Belgium). The assay was carried out according to the manufacturer’s instructions. Briefly, the multiplexed standard curve composed of the mixed cytokine standards was set up by serial dilutions. Selected capture beads were prepared and added to the standards and the supernatants. Following beads were used: TNFα (bead C4), IL-1α (bead D6), IL-1β (bead B4), IL-6 (bead A7), CCL2 (MCP1, bead D8), CCL3 (MIP1α, bead B9), CCL4 (MIP-1β, bead E4), CCL5 (RANTES, bead D4), and CXCL8 (IL-8, bead A9). After 1 h incubation, detection reagent was added to each sample and incubated for 2 h. Samples were rinsed with wash buffer, centrifuged, resuspended in wash buffer and analyzed by flow cytometry (BD FACSCanto II, BD Biosciences). Results were quantified using the Flow Cytometric Analysis Program Array software (Soft Flow, Minneapolis, MN, USA). To distinguish secretion induced by stimulation from spontaneous release by cell death, the concentrations found in the supernatants were normalized to lactate dehydrogenase (LDH) activity (Promega, Madison, WI, USA).

### Measurement of CCL4 and CXCL8 Secretion by ELISA

The secretion of CCL4 and CXCL8 in the supernatants was measured by ELISA bought from R&D systems. ELISAs were performed according to the manufacturer’s protocol and normalized to LDH.

### Statistics

Statistical analyses were performed using the GraphPad Prism software (Graph Pad Software, La Jolla, CA, USA). For time-course results with multiple stimulatory conditions, two-way ANOVA analysis was first performed to analyze the time and stimulation dependence of the results. Then a Tukey multiple comparison test was used to compare at each time point the effect of stimulation. For two-group comparison, normality and homogeneity of variances were ascertained, as determined by Kolmogorov–Smirnov and *F*-tests, respectively, and then Student’s *t*-test analyses were performed. Otherwise, Mann–Whitney tests were used for two-group comparisons. For all statistical tests, *p* < 0.05 was considered statistically significant. *p < 0.05; **p < 0.01; ***p < 0.001; ****p < 0.0001.

## Results

### S100A9 Is Released in a Phosphorylated Form

**Figure 1:**
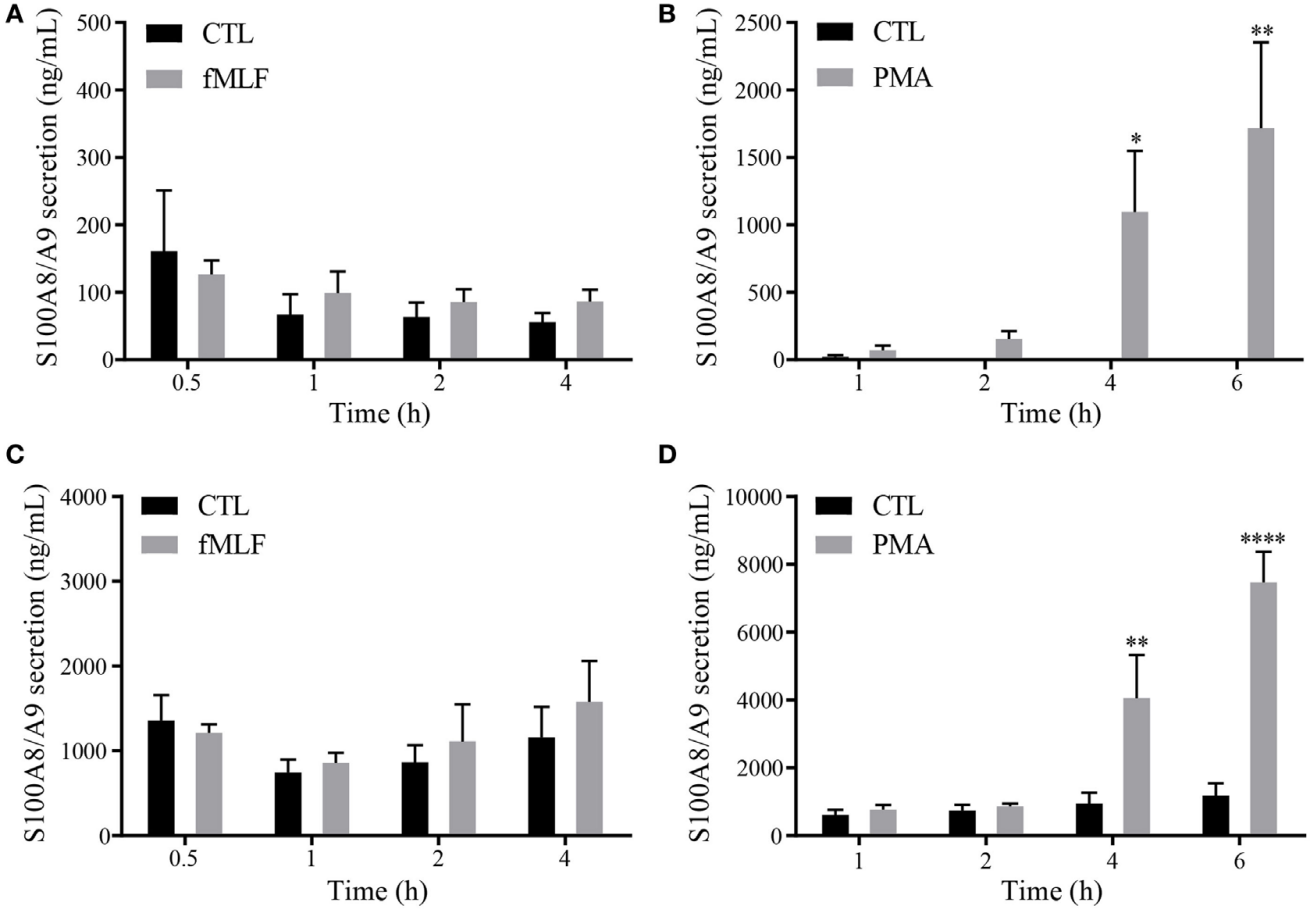
Secretion profile of S100A8/A9 under fMLF and phorbol 12-myristoyl 13-acetate (PMA) stimulation. Secretion of S100A8/A9 from differentiated HL-60 cells **(A,B)** or neutrophils **(C,D)** after stimulation with 100 nM fMLF **(A,C)** or 100 nM PMA **(B,D)** at different time points. S100A8/A9 concentrations were measured in cell supernatants by ELISA. Results are presented as mean ± SEM of five independent experiments. *p < 0.05; **p < 0.01; ***p < 0.001; ****p < 0.0001.

Since it is well established that PhosphoS100A9 contributes to microtubule reorganization (18) and regulation of the NADPH oxidase (5) at the intracellular level, we assume that the posttranslational modifications of S100A9 are also related to their inherent multiple extracellular functionalities. To confirm our hypothesis, we determined in a first step the phosphorylation state of S100A8/A9 secreted by neutrophil-like HL-60 cells (dHL-60) and purified neutrophils. In order to know under which stimulatory conditions S100A8/A9 are released in the cell culture supernatants, we performed a secretion time-course on dHL-60 cells or purified neutrophils stimulated with 100 nM fMLF or 100 nM PMA. Our results show no induction of S100A8/A9 secretion upon stimulation of dHL60 cells by fMLF (Figure 1A), while a 40-fold increased secretion is observed after 6 h of PMA addition (Figure 1B). Similar results were obtained on primary cells although the induction of S100A8/A9 secretion from PMA-stimulated neutrophils is more extensive than in dHL-60 cells (Figures 1C,D).

**Figure 2:**
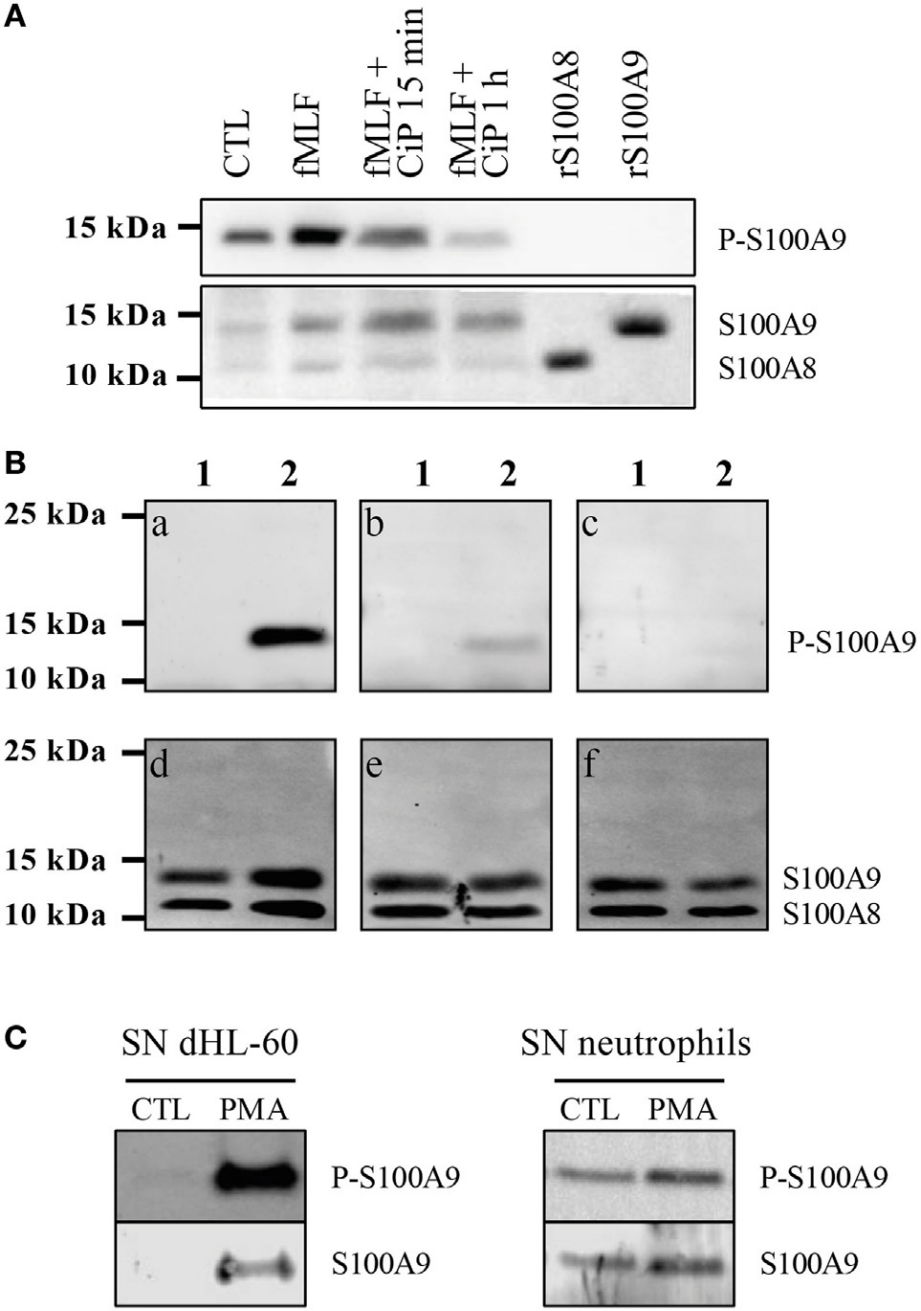
S100A9 is secreted under phosphorylated form. Validation of the custom-made phospho-specific S100A9 antibody **(A,B)**. **(A)** Non-stimulated cell lysates (CTL), fMLF-stimulated (100 nM, 10 min), phosphatase (CiP)-treated fMLF-stimulated cell lysates as well as recombinant S100A8 and S100A9 (2 μg) were run on a Tris-Tricine gel (10% acrylamide), transferred to PVDF membrane and probed with the anti-PhosphoS100A9 antibody and with the Mac387 antibody to detect total S100A8 and S100A9. **(B)** 3 μg of purified S100A8/A9 (lane 1) and S100A8/PhosphoA9 (lane 2) were run on a Tris-Tricine gel (10% acrylamide), transferred to PVDF membrane and probed with anti-PhosphoS100A9 antibody alone (a), preincubated 1 h with 1 μg/mL (b) or 4 μg/mL (c) of immunogenic peptide. The same membrane was also probed with B-5 and EPR3554 antibody in order to detect total S100A8 and S100A9 (d–f). **(C)** Supernatants (SNs) of control or PMA-stimulated dHL60 cells or purified neutrophils were concentrated with Centricon^®^ devices (cut-off 3 kDa) and then loaded on a 10% Tris-Tricine Gel. Western blots were probed with the anti-PhosphoS100A9 antibody and total S100A9 was detected by the Mac387 antibody.

In a second step, a polyclonal phospho-specific S100A9 antibody necessary to determine phosphorylation of secreted S100A9 was validated for specificity by Western Blot. Unphos-phorylated recombinant S100A8 and S100A9 were not recognized by the anti-P-S100A9 (Figure 2A). Stimulation of dHL-60 cells with fMLF, which induces phosphorylation of S100A9 by p38MAPK (5), led to the detection of a high signal at 14 kDa. Moreover, exposure of cell lysates to a phosphatase (CiP) for 15 min and 1 h, resulted in a time-dependent decrease of the anti-PhosphoS100A9 antibody signal, confirming the selectivity of this antibody for a phospho-protein at the same molecular weight as S100A9 (Figure 2A). Finally, we confirmed the specificity of anti-PhosphoS100A9 antibody by an antigen competition in western blot on purified S100A8/A9 and S100A8/PhosphoA9 proteins. A single band was detected in the S100A8/PhosphoA9 proteins lane (Figure 2B, a) which merged, in two-color fluorescence detection with the Odyssey system, with total S100A9 proteins (Figure 2B, d). Moreover, when anti-PhosphoS100A9 antibody was preincubated with an increasing concentration of immunogenic peptide, we observed a strong decrease and finally a total loss of the signal (Figure 2B, b and c). Taken together, these results demonstrate that the anti-PhosphoS100A9 antibody is specific for the phosphorylated form of S100A9 in western blot.

After having determined the conditions for S100A8/A9 secretion and controlled the selectivity of the anti-PhosphoS100A9 antibody, we investigated under which form S100A9 is found in the secreted S100A8/A9 complex. Thus, the supernatants of PMA-stimulated dHL-60 cells or purified neutrophils (Figure 2C) were concentrated and analyzed by Western Blot. Phospho-S100A9 and total S100A9 were detected concomitantly by the two-color fluorescence detection with the Odyssey system and our results clearly demonstrate that S100A9 is secreted as a phosphorylated form by dHL-60 cells and neutrophils.

### S100A8/A9 Release and NET Formation Are Linked in Neutrophils

**Figure 3:**
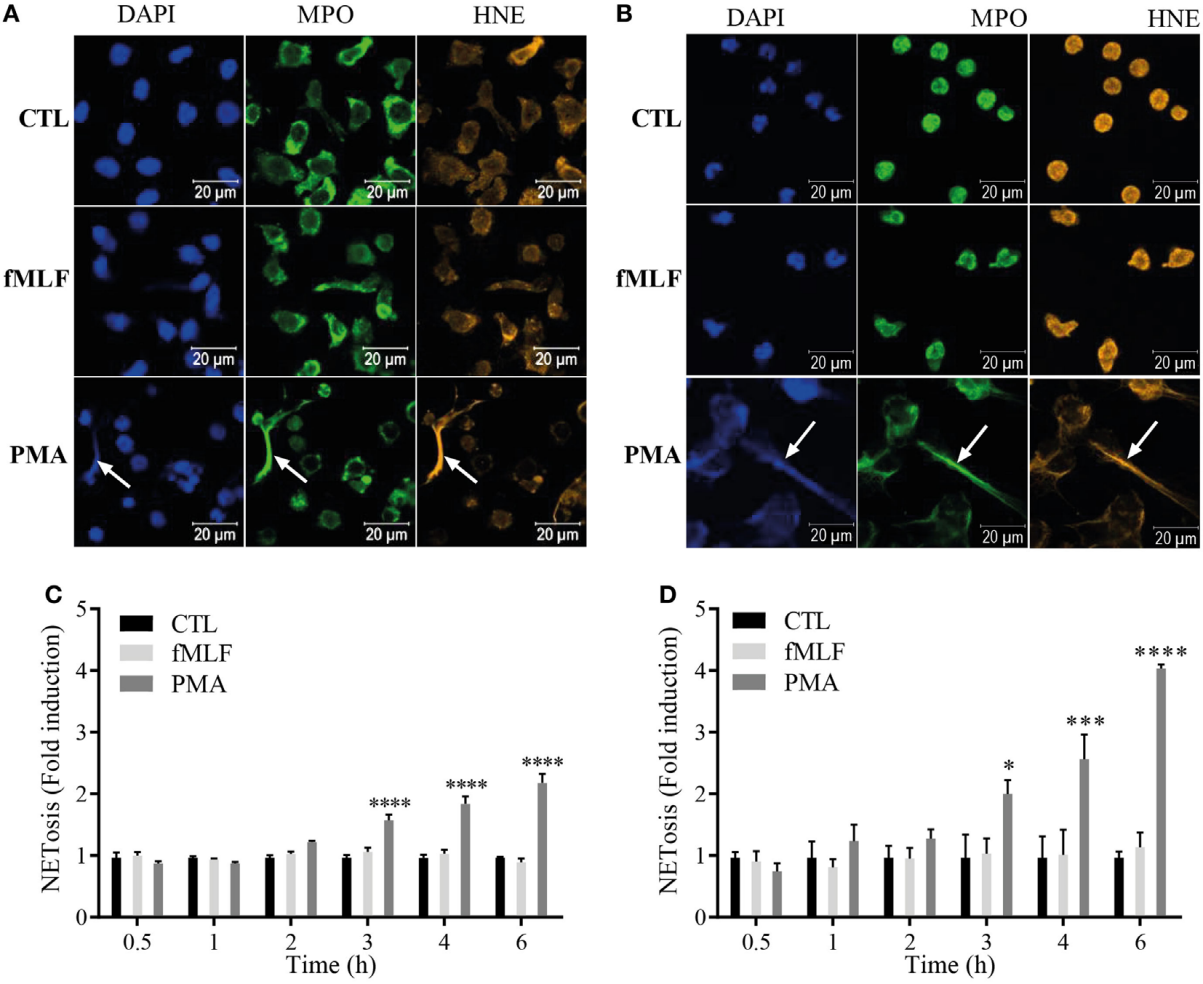
S100A8/A9 are secreted by NETosis from both differentiated HL-60 (dHL-60) cells and purified neutrophils. **(A,B)** Immunofluorescent staining of neutrophil extracellular traps (NETs) from dHL-60 cells **(A)** or neutrophils **(B)**. dHL-60 cells or neutrophils were stimulated with 100 nM fMLF (middle panels) or 100 nM phorbol 12-myristoyl 13-acetate (PMA) (lower panels) for 4 h and stained for DNA (DAPI; blue staining), myeloperoxidase (MPO; green staining) or neutrophil elastase (HNE; orange staining). Pictures are representative of at least three independent experiments. **(C,D)** Quantification of NET release from dHL60 cells **(C)** or neutrophils **(D)**. Extracellular NET-DNA was quantified with the cell-impermeable fluorescent dye Sytox blue and NET-release was detected *via* fluorescence emission. NET formation was normalized to the non-stimulated control and expressed as fold induction. Results are presented as mean ± SEM of four independent experiments. *p < 0.05; **p < 0.01; ***p < 0.001; ****p < 0.0001.

The secretion mechanism of S100A8/A9 from neutrophils is still not totally clarified and two hypotheses have been put forward: an active process dependent on an intact microtubule network (22) or a release by NETosis (23). Since we confirmed that S100A8/A9 is secreted by PMA stimulation and it is known that PMA is a typical NET inducer (32), we hypothesized that S100A8/A9 release from neutrophils may be mediated by NETosis. We visualized NET structures after 4 h of agonist stimulation in dHL60 cells (Figure 3A) and purified neutrophils (Figure 3B) by fluorescent staining of DNA, MPO, and human neutrophil elastase. The immunofluorescence images confirm that the cells, activated by PMA, release NETs as shown by the colocalization of extracellular DNA with MPO and neutrophil elastase. Cells stimulated by fMLF were not able to form NETs.

To compare the kinetics of NET formation to the ones of S100A8/A9 release, extracellular NET-DNA was monitored over time with the cell impermeant DNA dye Sytox blue in both dHL-60 cells (Figure 3C) and neutrophils (Figure 3D). PMA induces NET release from both dHL-60 cells (Figure 3C) and neutrophils (Figure 3D) beginning at 3 h of stimulation, while fMLF failed to induce NETs at any stimulation time point.

**Figure 4:**
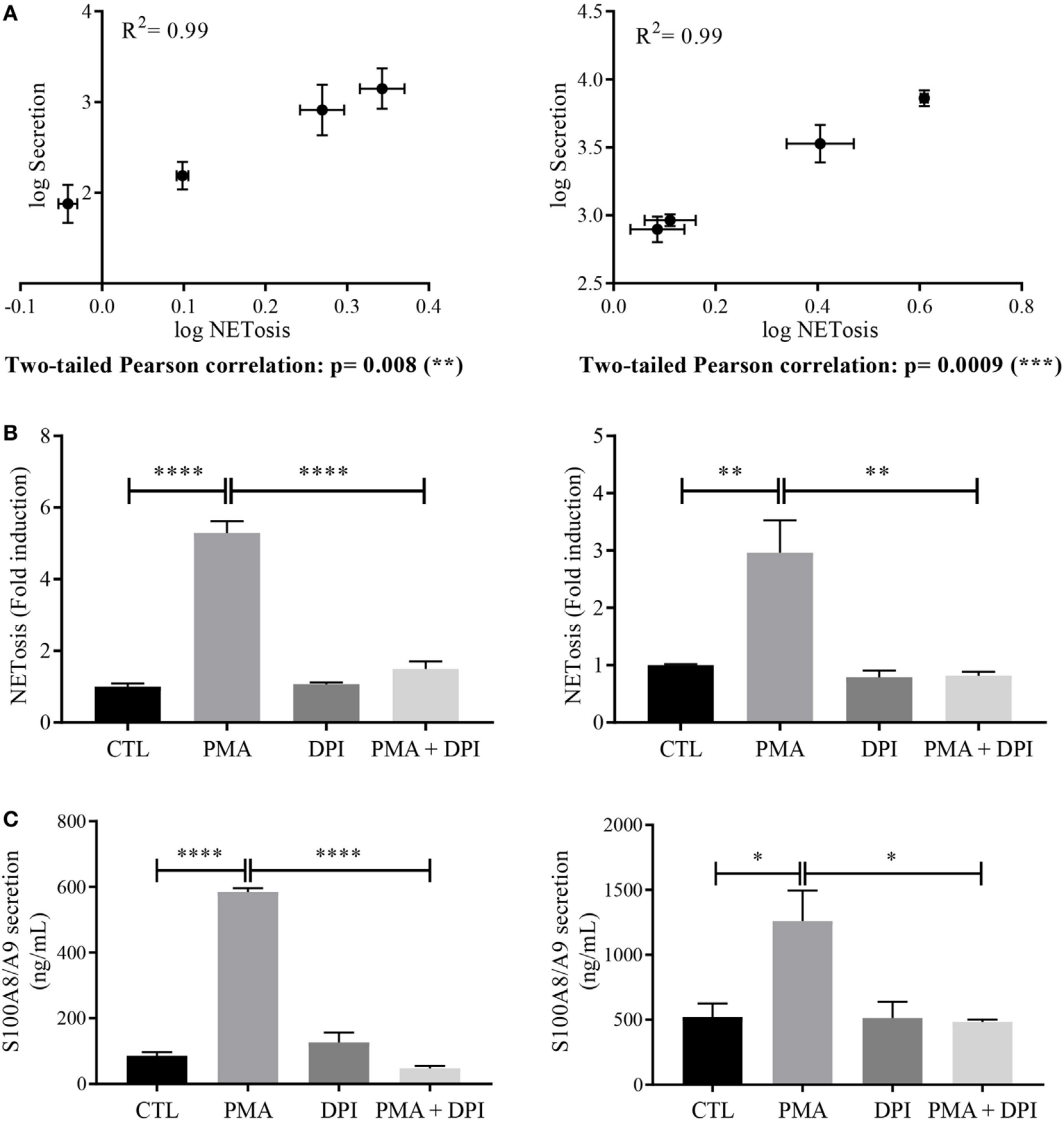
Relation between S100 secretion and neutrophil extracellular trap (NET) formation. **(A)** Correlation between NETosis and S100A8/A9 secretion. Log values of S100A8/A9 secretion were plotted against log values of NETosis and a two-tailed Pearson correlation test was performed for differentiated HL-60 (dHL-60) cells (left panel) or neutrophils (right panel). Involvement of nicotinamide adenine dinucleotide phosphate-oxidase (NADPH oxidase) in NETosis **(B)** and S100A8/A9 secretion **(C)** dHL-60 cells (left panel) or neutrophils (right panel) were incubated for 30 min with diphenyleneiodonium (10 μM) then stimulated with phorbol 12-myristoyl 13-acetate (PMA) (100 nM) for 6 h. S100A8/A9 concentration and extracellular NET-DNA were determined in the same condition than in Figures 1 and 3, respectively. Results are presented as mean ± SEM of three (for dHL60) or four (for neutrophils) independent experiments. *p < 0.05; **p < 0.01; ***p < 0.001; ****p < 0.0001.

These results underline the fact that S100A8/A9 are detected in the supernatants only when NETs are formed. By plotting S100A8/A9 secretion against NET formation and performing a Pearson correlation test, we obtained *p*-values of 0.008 and 0.0009 for dHL-60 (Figure 4A, left panel) and neutrophils (Figure 4A, right panel), respectively, supporting the assumption that S100A8/A9 secretion could be related to the NETosis process.

Since it is well established that ROS production is a hallmark of NET formation induced by PMA (33, 34), we inhibited the NADPH oxidase activation to strengthen the link between NETosis and S100A8/A9 secretion. For that, dHL-60 cells and neutrophils were treated for 30 min with 10 μM DPI before stimulation with PMA at 100 nM for 6 h.

As expected, in these conditions, NET formation in dHL-60 (Figure 4B, left panel) and neutrophils (Figure 4B, right panel) was blocked. More interestingly, DPI treatment also abolished PMA-induced S100A8/A9 secretion from both dHL-60 (Figure 4C, left panel) and neutrophils (Figure 4C, right panel).

Taken together these results strengthen our hypothesis that S100A8/A9 release could be dependent on NETosis process.

### S100A8/PhosphoA9, But not S100A8/A9, Induces Proinflammatory Cytokine mRNA Expression

**Figure 5:**
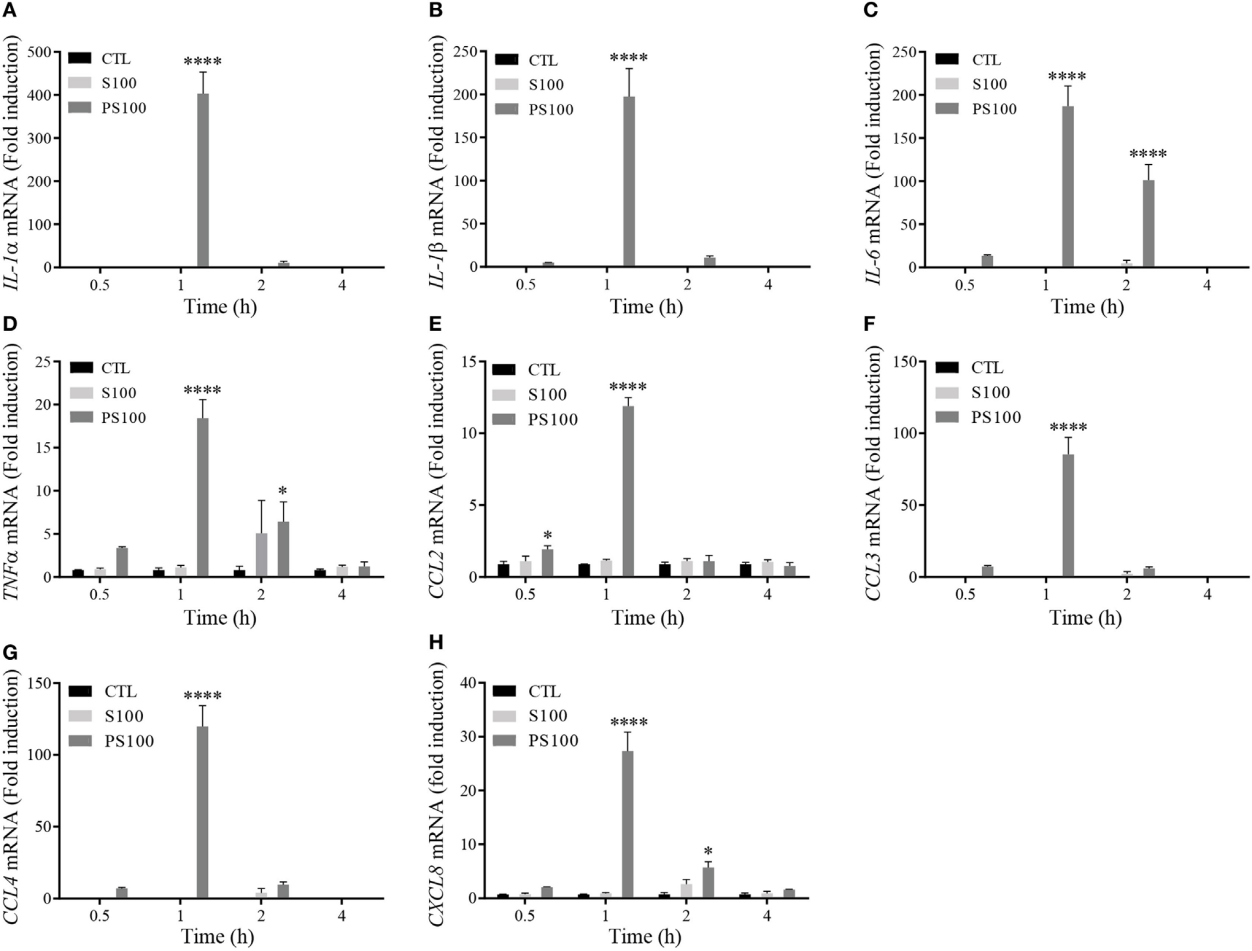
Cytokine mRNA expression after S100A8/A9 or S100A8/PhosphoA9 stimulation of differentiated HL-60 (dHL-60) cells. dHL-60 cells were stimulated for 30 min, 1, 2, and 4 h with 3 μg/mL of either S100A8/A9 or S100A8/PhosphoA9. Expression of *interleukin* (*IL)-1α* **(A)**, *IL-1β* **(B)**, *IL-6* **(C)**, *tumor necrosis factor alpha* (*TNFα*) **(D)**, *CCL2* **(E)**, *CCL3* **(F)**, *CCL4* **(G)**, and *CXCL8* **(H)** mRNA were measured by real-time PCR. Results were normalized to three reference genes (*Actin-β, B2M*, and *Gusβ*) according to the method of Vandesompele et al. (31) and expressed as fold induction compared to the non-stimulated control (22). Results are presented as mean ± SEM of three independent experiments. *p < 0.05; **p < 0.01; ***p < 0.001; ****p < 0.0001.

To confirm our initial hypothesis on the proinflammatory function of extracellular PhosphoS100A9, we decided to monitor the effects of unphosphorylated and phosphorylated S100A9 on cytokine/chemokine expression, as these mediators are essential for the development and regulation of immune processes and inflammation. dHL-60 cells were left untreated (CTL) or incubated in presence of 3 μg of either S100A8/A9 (S100) or S100A8/PhosphoA9 (PS100) for 0.5, 1, 2, or 4 h, then the mRNA levels of the proinflammatory cytokines *IL-1α, IL-1β, IL-6*, and *TNFα* and the chemokines *CCL2, CCL3, CCL4*, and *CXCL8* were quantified (Figure 5). No cytokine or chemokine mRNA expression was induced by S100A8/A9, while dHL-60 cells responded to S100A8/PhosphoA9 stimulation by a significant induction of all tested cytokines and chemokines (ranging from 12-fold induction for *CCL2* to a 400-fold induction for *IL-1α*). The induction reached a maximum after 1 h of stimulation and decreased to basal levels after 2 h of stimulation. Only *IL-6, TNFα* and *CXCL8* displayed elevated levels at 2 h. In addition, cytokine expression varied in magnitude, with *IL-1α, IL-1β, IL-6, CCL3*, and *CCL4* displaying a high differential expression (induction higher than 75-fold after S100A8/PhosphoA9 stimulation), whereas *TNFα, CCL2*, and *CXCL8* showed a more moderate induction. To exclude an endotoxin contamination in the Phospho-S100 preparations, a Limulus amebocyte assay was performed. However, this assay indicated that one of the S100A8/PhosphoA9 batches used had an endotoxin contamination of 0.1 ng/mL, which alone did not induce any cytokine mRNA expression after 1 h of stimulation. Results obtained by a non-contaminated S100A8/PhosphoA9 batch showed similar results to the contaminated batch and a costimulation of dHL-60 cells with LPS and S100A8/A9 did not induce cytokine expression either (Figure S1 in Supplementary Material). Moreover, heat inactivation (30 min, 80°C) of S100A8/PhosphoA9 triggered a strong inhibition of RNA expression of cytokines (Figure S2 in Supplementary Material). Altogether, these results indicate that the induction of the various cytokine mRNA we observed with the S100A8/PhosphoA9 is not influenced by the small endotoxin contamination, revealing that only S100A8/PhosphoA9 is functionally active on neutrophil-like dHL-60 cells and that it induces a quite rapid and short response.

### S100A8/PhosphoS100A9 Induces the Release of Proinflammatory Cytokines

**Figure 6:**
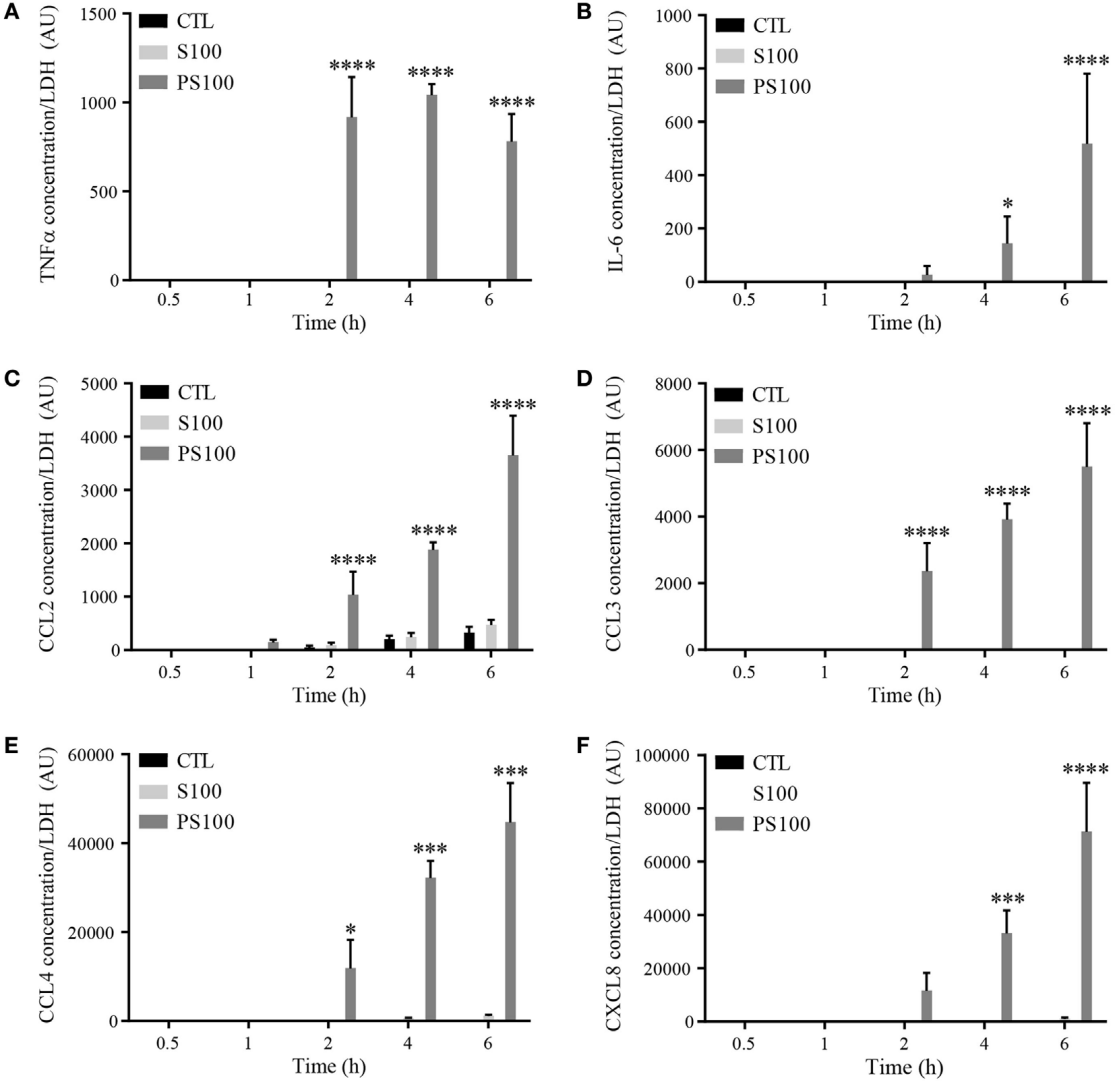
Time-dependent cytokine secretion from differentiated HL-60 cells after S100A8/A9 or S100A8/PhosphoA9 stimulation. Cytokine secretion was measured by cytometric bead array upon stimulation with 3 μg/mL S100A8/A9 or S100A8/PhosphoS100A9 for 30 min to 6 h. Tumor necrosis factor alpha (TNFα) **(A)**, interleukin (IL)-6 **(B)**, CCL2 **(C)**, CCL3 **(D)**, CCL4 **(E)**, or CXCL8 **(F)** concentrations were divided by LDH values in order to correct for cytokine release by cell death. Results are presented as mean ± SEM of three independent experiments. *p < 0.05; **p < 0.01; ***p < 0.001; ****p < 0.0001.

mRNA levels do not necessarily correlate to the relative amount of proteins released. We thus measured the secretion of proinflammatory cytokines upon S100A8/A9 or S100A8/PhosphoA9 stimulation to appreciate the functional effect of extracellular S100A9 phosphorylation. dHL-60 cells were treated with 3 μg/mL of S100A8/A9 or S100A8/PhosphoA9 for 30 min and up to 6 h; the presence of the different cytokines and chemokines in the supernatants was measured by multiplexed CBA. The cytokines/chemokines tested were the ones found to be induced on the mRNA levels by S100A8/PhosphoA9 stimulation, namely *IL-1α, IL-1β, TNFα, IL-6, CCL2, CCL3, CCL4*, and *CXCL8* (Figure 6). Although *IL-1α, IL-1β* mRNA expression was induced in S100A8/PhosphoA9-stimulated cells, no secretion was detected in any stimulatory condition. TNFα, CCL2, CCL3, and CCL4 were secreted already after 2 h of S100A8/PhosphoA9 stimulation and IL-6 and CXCL8 were released at 4 and 6 h of S100A8/PhosphoA9 stimulation (Figure 6). CCL4 and CXCL8 secretion was highly induced while the release of TNFα, IL-6, CCL2, and CCL3 was more moderate. Stimulation with S100A8/A9 induced no cytokine secretion at any time point, confirming that the phosphorylated form S100A8/PhosphoA9 is the active form of the heterocomplex. As indicated previously, to exclude an effect of the LPS contamination on S100A8/PhosphoA9-induced cytokine secretion, dHL-60 cells were stimulated with 0.1 ng/mL LPS for 4 h, and CXCL8 and CCL4 secretion was measured by ELISA. No CXCL8 or CCL4 secretion was detected after LPS stimulation, while the secretion was highly induced by S100A8-phosphoA9 in the same conditions (Figure S3 in Supplementary Material). Moreover, S100A8/PhosphoA9-induced effects could not be inhibited by incubation with the LPS inhibitor Polymyxin B at concentrations which inhibited by 75% the secretion of CXCL8 and CCL4 induced by 100 ng/mL LPS (Figure S3 in Supplementary Material). Thus, the S100A8/PhosphoA9 complex has the functional role in the induction of cytokine release from dHL-60 cells.

### S100A8/PhosphoS100A9 Affect Proinflammatory Cytokine Release through TLR4 Signaling

**Figure 7:**
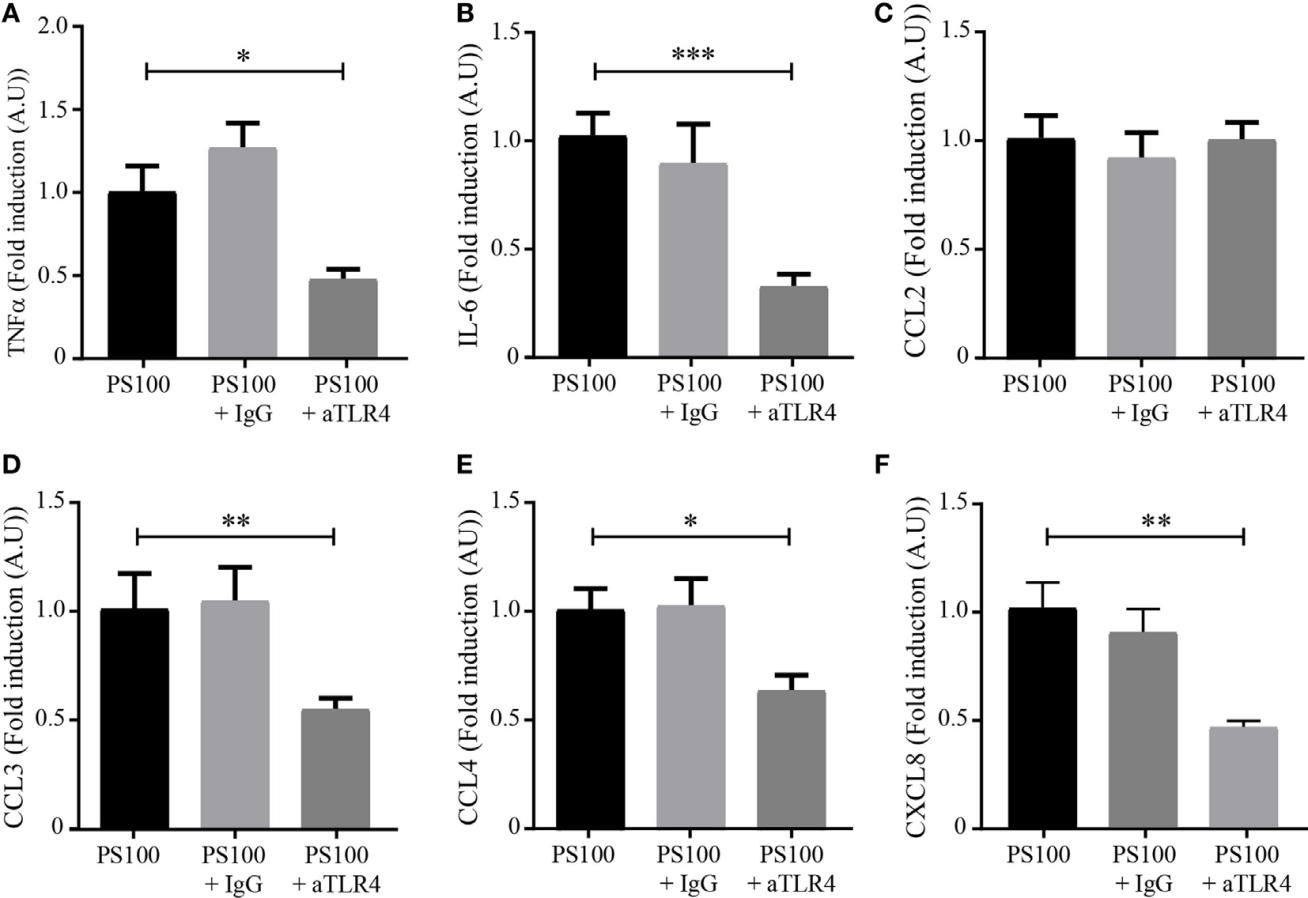
TLR4-dependence of S100A8/PhosphoA9-induced cytokine secretion. Differentiated HL-60 cells were incubated with 1 μg/mL of neutralizing toll-like receptor 4 (TLR4) antibody (aTLR4) or isotype control (IgG) and stimulated with 3 μg/mL S100A8/PhosphoA9 (PS100) for 4 h. Cytokine secretion was measured by cytometric bead array. Concentrations for tumor necrosis factor alpha (TNFα) **(A)**, interleukin (IL)-6 **(B)**, CCL2 **(C)**, CCL3 **(D)**, CCL4 **(E)**, or CXCL8 **(F)** were divided by LDH values and the results expressed as fold induction of the S100A8/PhosphoA9-stimulated control. Results are presented as mean ± SEM of five independent experiments. *p < 0.05; **p < 0.01; ***p < 0.001; ****p < 0.0001.

Toll-like receptor 4 was described to be important for S100A8/A9 signaling in various cell types (2, 35). In this view, we investigated the involvement of TLR4 signaling in the S100A8/PhosphoA9-mediated response, by using a TLR4 neutralizing antibody. TLR4 was blocked with 1 μg/mL of neutralizing antibody, dHL-60 cells were stimulated with 3 μg/mL S100A8/PhosphoA9 for 4 h and secretion of TNFα, IL-6, CCL2, CCL3, CCL4, and CXCL8 was measured. Results clearly show a reduced secretion for TNFα, IL-6, CCL4, and CXCL8 when TLR4 was blocked, while CCL2 secretion was not affected at all (Figure 7). The incubation of dHL60 cells with a goat control IgG and stimulated with S100A8/PhosphoA9 showed no difference in cytokine secretion when compared to the S100A8/PhosphoA9 stimulated cells (data not shown). Taken together, these results suggest that TLR4-mediated signaling pathways are involved in S100A8/PhosphoA9-induced cytokine secretion in dHL60 cells, but also that another/other receptor(s)/partner(s) seem(s) to participate in S100A8/PhosphoA9-induced CCL2 secretion.

## Discussion

While the role of S100A8/A9 in chronic inflammatory diseases and tumorigenesis has been increasingly recognized in the literature, our understanding of the molecular mechanisms underlying their involvement in such pathologies is just at the beginning. The discovery of the phosphorylation of intracellular S100A9 by p38 MAPK raises the possibility that the posttranslational modification of S100A9 is associated with the proinflammatory functions of S100A8/A9 heterocomplexes, potentially allowing diversification of its functions. In accordance with this assumption, we found that S100A9 is secreted in a phosphorylated form by neutrophils.

The mechanism by which S100A8 and S100A9 are secreted is still elusive, but it has become apparent over the years that the mode of secretion of these proteins is cell-type specific. In monocytes different stimuli such as PMA, LPS, or IL-1β induce an energy and PKC-dependent process relying on an intact microtubule network (22). In the same way, interaction of monocytes with activated endothelial cells induces a calcium-dependent secretion of S100A8/A9 (36). The difference between S100A8/A9 secretion by monocytes and neutrophils was identified in human phagocytes activated by monosodium urate monohydrate (MSU) crystals, which are largely used to induce a peritoneal model of acute gout. In this model it was found that S100A8/A9 release from neutrophils was dependent on cell death, while S100A8/A9 were actively secreted from undamaged monocytes (37). Such a difference could be explained by the fact that MSU crystals induce NET formation from neutrophils but not from monocytes (38).

**Figure 8:**
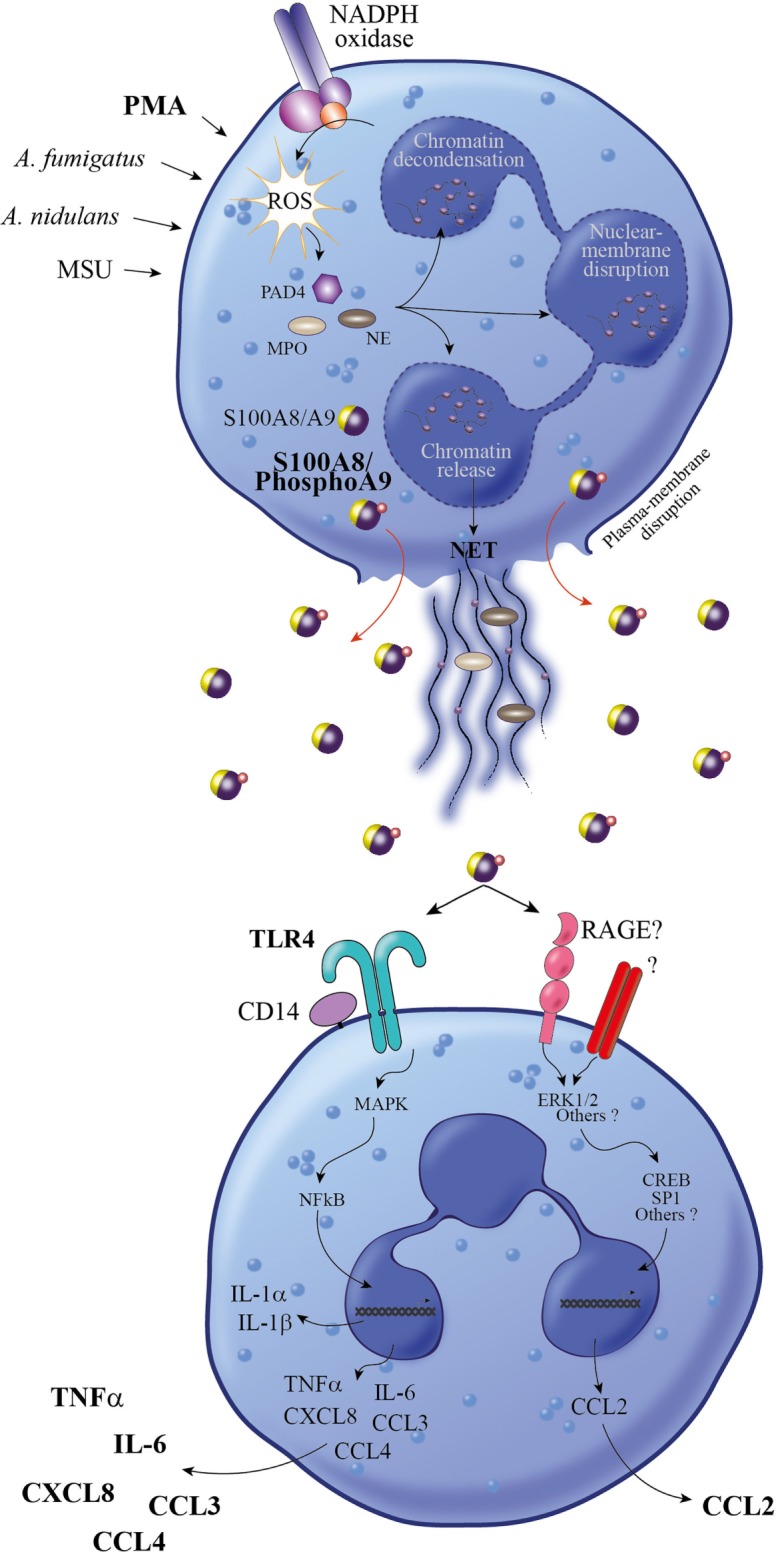
Proposed model for the proinflammatory response induced by S100A8/PhosphoA9. Neutrophils activated under different stimulatory conditions (PMA, MSU, *Aspergillus fumigates*, or *Aspergillus nidulans*) are releasing NETs through a mechanism involving NADPH oxidase, PAD4, NE, and MPO (34). During NET formation, active S100A8/A9 are released in the extracellular space in a phosphorylated form. S100A8/PhosphoA9 can then activate surrounding neutrophils to secrete cytokines (TNFα and IL-6) and chemokines (CCL2, CCL3, CCL4, and CXCL8). This release is mainly regulated through TLR4 signaling pathways, although other receptors (such as RAGE) are involved in S100A8/PhosphoA9-mediated CCL2 secretion. Thus, S100A8/PhosphoA9 released from neutrophils are involved in the amplification of the inflammatory process and could be the hallmark of various inflammatory diseases. MPO, myeloperoxidase; MSU, monosodium urate monohydrate; NE, neutrophil elastase; PAD4, peptidyl arginine deiminase; PMA, phorbol 12-myristoyl 13-acetate; NET, neutrophil extracellular trap; NADPH oxidase, nicotinamide adenine dinucleotide phosphate-oxidase; TNFα, tumor necrosis factor alpha; IL-6, interleukin 6.

Initially, NETs have been described to have an important direct microbicidal function, but meanwhile, their importance as signaling cues in modulating the inflammatory response and alerting the immune system to infection by releasing DAMPs has also been recognized (32, 39–41). In this regard, S100A8/A9 have been detected in association with NETs in neutrophils which were induced by *Aspergillus fumigatus* (42) or *Aspergillus nidulans* (23) stimulation. Our results indicate that secretion of S100A8/A9 upon PMA stimulation is correlated with the formation of NETs, supporting the fact that S100A8/A9 are released by NETosis from neutrophils. There is a growing awareness that multiple processes are involved in NET formation, including generation of ROS by the NADPH oxidase (33), histone citrullination, actin filamentation, and microtubule polymerization (43, 44) (Figure 8). Since the phosphorylation of S100A9 was described as a regulator of the S100A8/A9-microtubule interaction (26), it is tempting to speculate that Phospho-S100A9 supports NETosis and thus, effective secretion of S100A8/PhosphoA9 from neutrophils.

A recent study provided evidence for a role of S100A8 and S100A9 in the secretion of IL-6, TNFα, and IL-1β through the production of ROS, which activates the transcription factor NF-κB in peripheral blood mononuclear cells (45). The same group reported that human monocytes stimulated by recombinant S100A9 were able to dose-dependently secrete IL-6 and TNFα, but not neutrophils (46). According to our previous data, we questioned whether phosphorylation of S100A9 in the secreted S100A8/A9 complex could bear the proinflammatory potential of neutrophils. We thus analyzed the effect of S100A8/PhosphoA9 on cytokine and chemokine secretion from dHL-60 cells. Indeed, since a small percentage of monocyte contamination, as low as 1%, can result in a significant increase in cytokine expression compared to a pure neutrophil fraction (47), dHL-60 cells represent a pure adequate model to investigate the role of S100A8/PhosphoA9 on neutrophil cytokine secretion. Cytokines have been selected according to the secretion profile previously established upon inflammatory conditions induced by LPS (48), which is known to bind to pattern recognition receptors (TLR4).

In fact, S100A8/PhosphoA9 was able to induce the expression of *TNFα, IL-1α, IL-1β, IL-6, CCL2, CCL3, CCL4*, and *CXCL8*, but IL-1α and IL-1β were not secreted. In this sense, it seems that an accumulation of cytokine mRNA does not necessarily lead to an increased secretion as previously claimed (47). However, IL-1α and IL-1β could be stored in neutrophils in order to be rapidly mobilizable upon a subsequent stimulation. This is in line with the fact that cytokine release is insured by a spatiotemporal regulation enabling the development of an appropriate inflammatory response.

S100A8/PhosphoA9 trigger the release of chemotactic factors for neutrophils (CXCL8), monocytes (CCL2), and natural killer cells (CCL3, CCL4) [for review see Ref. (49)] as well as of TNFα which can exert a potent autocrine effect by amplifying the production of neutrophil-derived cytokines and chemokines (50). Therefore, extracellular S100A8/PhosphoA9 may orchestrate the recruitment of immune cells to the site of inflammation and also contribute to the persistence of the inflammatory response.

Our finding is thus pointing to the phosphorylation of S100A9 as an additional regulatory level in the induction of cytokine expression and secretion in neutrophil-like cells (Figure 8). This phosphorylation could indeed be a prerequisite for the activation of neutrophils by the S100A8/A9 complex, which may explain why no cytokine secretion could be measured on neutrophils triggered using recombinant, thus unphosphorylated, S100A9 (46, 51). As a consequence, recombinant S100A9 should not be used to investigate the proinflammatory functions of S100A8/A9 on neutrophils.

We also show that cytokine secretion induced by S100A8/PhosphoA9 involves TLR4-mediated signaling pathways, in accordance with previous studies demonstrating that TLR4 has a dominant role during S100A8-mediated phagocyte activation (52) and S100A9-mediated cytokine induction in THP-1 cells (53). However, the release of CCL2 was not decreased by TLR4 inhibition in our experimental conditions, supporting the assumption that distinct receptors, such as for example RAGE and downstream signaling pathways may be activated by S100A8/PhosphoA9 to ensure an appropriate inflammatory response (Figure 8).

Our results contribute to the demonstration that S100A8 and S100A9 are critical for an efficient proinflammatory process but are also key actors of the development of many chronic inflammatory diseases and cancer. Further studies are needed to provide a complete overview of the multifaceted proinflammatory functions of S100A8/PhosphoA9 including identification of signaling pathways induced which could be intently pursued targets for therapeutic intervention.

## Ethics Statement

Human neutrophil samples from healthy subjects were collected under written informed consent and following the good clinical and ethical practices approved by both the Ethics Review Panel (ERP) of the University of Luxembourg and the “Comité National d’Ethique de Recherche” (CNER) from Luxembourg.

## Author Contributions

VS, SP, NJ, JH, and FT performed experiments; FT, ET, and VS had substantial contributions to the conception and design of the work; VS, SB, and FT wrote the article; SP, J-LB, SB, ET, and FT revised critically the work.

## Conflict of Interest Statement

The authors declare that the research was conducted in the absence of any commercial or financial relationships that could be construed as potential conflict of interest.
