# Supplementary material for: Secretion of the Phosphorylated Form of S100A9 from Neutrophils Is Essential for the Proinflammatory Functions of Extracellular S100A8/A9
Source: Front Immunol. 2018 Mar 13;9:447. doi: 10.3389/fimmu.2018.00447 (PMC5859079; doi:10.3389/fimmu.2018.00447)
Supplement: Supplementary file 1 [file Data_Sheet_1.DOCX]

Supplementary Material

Secretion of the phosphorylated form of S100A9 from neutrophils is essential for the pro-inflammatory functions of extracellular S100A8/A9

Véronique Schenten, Sébastien Plançon, Nicolas Jung, Justine Hann, Jean-Luc Bueb, Sabrina Bréchard, Eric J. Tschirhart and Fabrice Tolle*

*** Correspondence:** Fabrice Tolle, Calcium Signalling and Inflammation Laboratory, Life Sciences Research Unit, University of Luxembourg, L-4367 Belvaux, Luxembourg. Mail to: fabrice.tolle@uni.lu

# Supplementary Figures

##
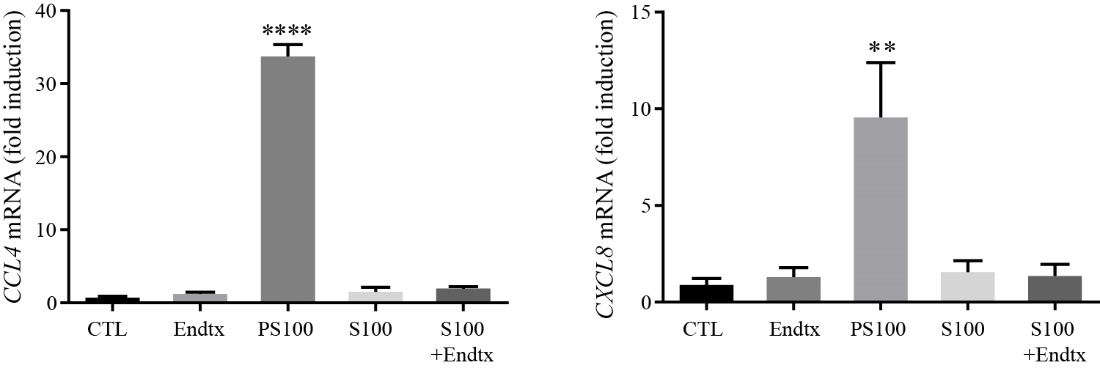
Supplementary Figure 1

**Supplementary Figure 1.** Effect of endotoxin in S100A8/PhosphoA9 preparation on cytokines RNA expression. Differentiated HL-60 cells were stimulated for 1 hour by *Escherichia coli* endotoxin (Endtx, 0.1 ng/mL), S100A8/PhosphoA9 (PS100, 3 μg/mL), S100A8/A9 (S100, 3 µg/mL) and S100A8/A9 plus *Escherichia coli* endotoxin (S100+Endtx, 3 µg/mL and 0.1 ng/mL, respectively). The mRNA levels of *CXCL8* and *CCL4* were quantified by qPCR. Results are presented as mean ± SEM of 3 independent experiments. * = p < 0.05; ** = p < 0.01; *** = p < 0.001; **** = p < 0.0001.

##
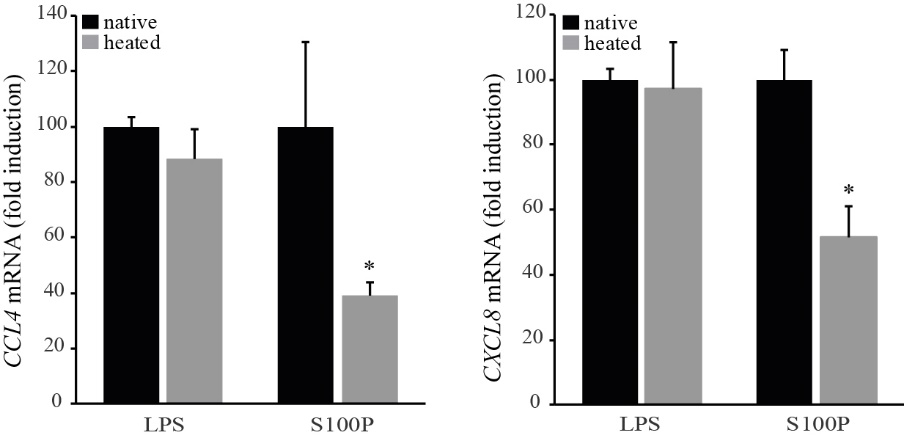
Supplementary Figure 2

**Supplementary Figure 2.** Effect of S100A8/PhosphoA9 heat inactivation on RNA expression of cytokines. Differentiated HL-60 cells were stimulated for 1 hour by LPS (100 ng/mL) or S100A8/PhosphoA9 (3 μg/mL) on their native form or after heating (30 min at 80°C). The mRNA levels of *CXCL8* and *CCL4* were quantified by qPCR. Results are presented as mean ± SEM of 3 independent experiments. * = p < 0.05; ** = p < 0.01; *** = p < 0.001; **** = p < 0.0001.

##
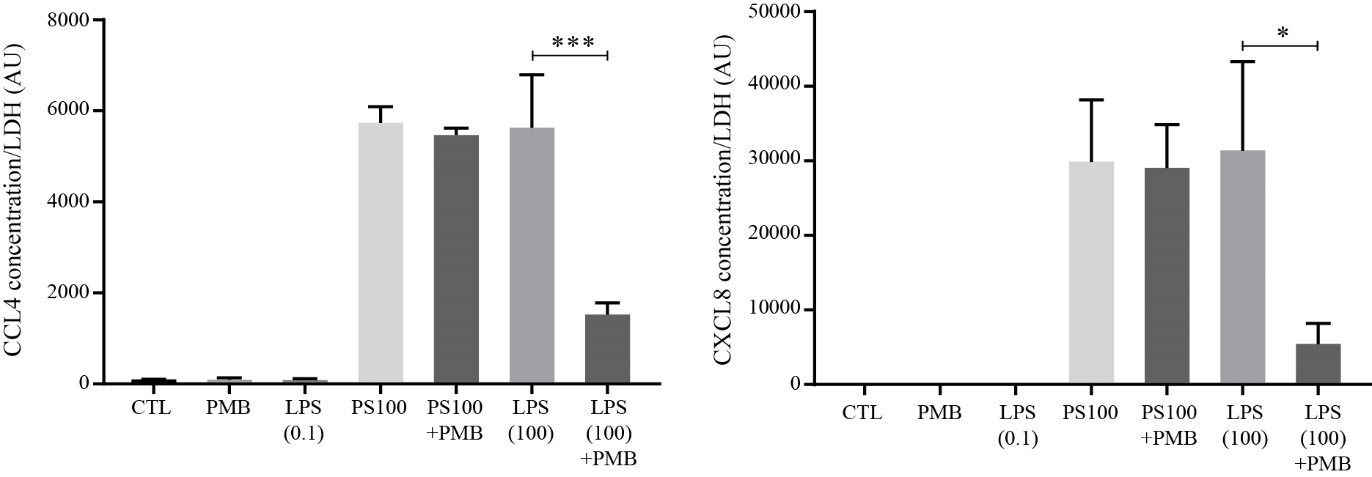
Supplementary Figure 3

**Supplementary Figure 3.** Effect of endotoxin in S100A8/PhosphoA9 preparation on cytokine secretion. CCL4 and CXCL8 secretion was measured by ELISA upon stimulation for 4 hours of differentiated HL-60 cells by Polymyxin B (PMB, 10 µM), lipopolysaccharide (LPS) at 0.1 ng/mL (LPS0.1) or 100 ng/mL (LPS100), S100A8/PhosphoA9 (PS100, 3 μg/mL) alone or in combination. CCL4 and CXCL8 concentration were divided by LDH values in order to correct for cytokine release by cell death. Results are presented as mean ± SEM of 3 independent experiments. * = p < 0.05; ** = p < 0.01; *** = p < 0.001.
